# Participation of Dopamine D1 and D2 Receptors in the Rapid-Onset Behavioral Sensitization to Modafinil

**DOI:** 10.3389/fphar.2019.00211

**Published:** 2019-03-11

**Authors:** Raphael Wuo-Silva, Daniela F. Fukushiro-Lopes, Bruno P. Fialho, André W. Hollais, Renan Santos-Baldaia, Eduardo A. V. Marinho, Elisa Mári-Kawamoto, Thaís S. Yokoyama, Leonardo B. Lopes-Silva, Laís F. Berro, Roberto Frussa-Filho, Beatriz M. Longo

**Affiliations:** ^1^Laboratory of Neurophysiology, Department of Physiology, Universidade Federal de São Paulo, São Paulo, Brazil; ^2^Department of Pharmacology, Universidade Federal de São Paulo, São Paulo, Brazil; ^3^Department of Health Sciences, Universidade Estadual de Santa Cruz, Ilhéus, Brazil; ^4^Department of Psychiatry and Human Behavior, University of Mississippi Medical Center, Jackson, MS, United States

**Keywords:** mice, modafinil, SCH 23390, sulpiride, rapid-onset behavioral sensitization

## Abstract

Studies on the abuse potential of modafinil, a psychostimulant-like drug used to treat narcolepsy, are still controversial. While some studies claim no potential for abuse, increasing evidence suggests that modafinil induces abuse-related effects, including rapid-onset behavioral sensitization (i.e., a type of sensitization that develops within hours from the drug priming administration). The rapid-onset sensitization paradigm is a valuable tool to study the neuroplastic changes that occur quickly after drug administration, and shares neuroadaptations with drug abuse in humans. However, the mechanisms involved in the rapid-onset behavioral sensitization induced by modafinil are uncertain. Our aim was to investigate the possible involvement of dopamine D1 and D2 receptors on acute modafinil-induced hyperlocomotion and on the induction and expression of rapid-onset behavioral sensitization induced by modafinil in male Swiss mice. Treatment with the D1 receptor antagonist SCH 23390 or the D2 receptor antagonist sulpiride attenuated the acute modafinil-induced hyperlocomotion in a dose-dependent manner. Pretreatment with either antagonist before the priming injection of modafinil prevented the development of sensitization in response to a modafinil challenge 4 h later. However, only SCH 23390 decreased the expression of modafinil-induced rapid-onset behavioral sensitization. Taken together, the present findings provide evidence of the participation of D1 and D2 receptors on the development of rapid-onset behavioral sensitization to modafinil, and point to a prominent role of D1 receptors on the expression of this phenomenon.

## Introduction

Modafinil (diphenyl-methyl sulphonyl-2-acetamide) is a wake-promoting drug with psychostimulant properties that has been approved for the treatment of excessive daytime sleepiness in narcolepsy, obstructive sleep apnea and shift workers sleep disorder (Minzenberg and Carter, 2008). Modafinil also shows potential benefits for the treatment of psychiatric and neurologic disorders, including attention-deficit/hyperactivity disorder, cognitive deficits related to schizophrenia and Alzheimer’s disease, sleepiness and fatigue related to Parkinson’s disease and amyotrophic lateral sclerosis (Ballon and Feifel, 2006; Minzenberg and Carter, 2008; Minzenberg et al., 2018). Although studies have reported a limited potential for abuse of modafinil (Deroche-Gamonet et al., 2002; Vosburg et al., 2010; Shuman et al., 2012; Uguen et al., 2013), this subject is still controversial.

Modafinil is a drug with multiple mechanisms of action, acting on a broad spectrum of neurotransmitter systems, including catecholamines, glutamate, GABA, serotonin, histamine and orexin (Ballon and Feifel, 2006; Minzenberg and Carter, 2008). Similar to other psychostimulant drugs that exert abuse liability in humans, such as cocaine, modafinil blocks dopamine transporters (DAT), thereby increasing the extracellular concentration of dopamine in the nucleus accumbens (Volkow et al., 2009; Funayama et al., 2014). Also similarly to other drugs of abuse, modafinil has been shown to produce withdrawal symptoms upon discontinuation of use (Krishnan and Chary, 2015). Bernardi et al. (2009) have demonstrated that modafinil at high doses reinstated cocaine-induced conditioned place preference following extinction in rats, suggesting that modafinil may increase the incentive salience of drug-related environmental cues. Moreover, modafinil alone also seems to exert rewarding properties, as it produces conditioned place preference and induces robust behavioral sensitization after single- and repeated-injection treatments in mice (Paterson et al., 2010; Wuo-Silva et al., 2011; Shuman et al., 2012). More recently, modafinil has been shown to induce rapid-onset behavioral sensitization in mice using a paradigm in which sensitization is developed when a challenge drug injection is administered only a few hours after a priming injection of a high dose of the same drug (Wuo-Silva et al., 2016).

Behavioral sensitization has been used to study the neurochemical mechanisms involved in the dopaminergic mesoaccumbens plasticity that are thought to play a major role in the reinforcing effects, incentive salience, and craving induced by drugs of abuse in humans (Robinson and Berridge, 1993, 2008; Vezina et al., 2007). The mechanisms involved in behavioral sensitization are thought to be related, at least in part, to long-lasting changes in dopamine D1 and D2 receptors localized in the ventral tegmental area (VTA) and in the nucleus accumbens (Henry and White, 1991; Henry et al., 1998; Camarini et al., 2011). Such events result in long-lasting neuroadaptations in the mesolimbic dopaminergic system that are thought to be responsible for the development and expression of behavioral sensitization (Bibb et al., 2001; Hu et al., 2002).

Considering the importance of the dopamine mesolimbic system for psychostimulant-induced behavioral sensitization, in this study we aimed to investigate the possible involvement of dopamine D1 and D2 receptors in the induction and the expression of modafinil-induced rapid-onset behavioral sensitization in mice. We administered the D1 receptor antagonist SCH 23390 or the D2 receptor antagonist sulpiride at different phases of behavioral sensitization to modafinil.

## Materials and Methods

### Subjects

Male 3-month-old Swiss EPM-M2 mice (40–45 g) from our own colony were used. Animals were housed in polypropylene cages (33 cm × 44 cm × 17 cm) under conditions of controlled temperature (22–23°C) and lighting (12/12 h light/dark, lights on at 06:45 h). Food and water were available *ad libitum* throughout the experiments. Each cage contained animals from the same experimental group.

The experimental protocols were approved by the Institutional Animal Care and Use Committee of UNIFESP/SP (Universidade Federal de São Paulo, UNIFESP – #8030060514). All animals were housed in a pathogen-free facility and were maintained in accordance with the National Institute of Health Guide for the Care and Use of Laboratory Animals (NIH Publications No. 8023), revised in 2011. All measures were taken to minimize pain and discomfort throughout the study.

### Drugs

Modafinil (64 mg/kg, CEPHALON^®^, Maisons-Alfort, France) was dissolved in 0.5% gum arabic and diluted in 0.9% NaCl (saline) solution. Sulpiride (25, 50, and 100 mg/kg, Sigma-Aldrich, São Paulo, Brazil) was dissolved in tween 80 and diluted in 0.9% NaCl (saline) solution. SCH 23390 (0.003, 0.006, and 0.01 mg/kg, Sigma-Aldrich, São Paulo, Brazil) was freshly diluted in 0.9% NaCl (saline) solution. Modafinil vehicle, sulpiride vehicle and saline were used as control (Veh) solutions. The solutions were administered intraperitoneally (i.p.) at a volume of 10 ml/kg body weight. The doses of the dopamine antagonists used in this study were based on the literature (Kuribara, 1995; Cervo and Samanin, 1996; Le Merrer and Stephens, 2006; Fish et al., 2014) and in previous studies conducted by our group (unpublished data). We chose doses of dopaminergic antagonists that did not alter spontaneous locomotion in mice, yet had been shown to decrease behavioral sensitization induced by other psychostimulant drugs. The dose of modafinil (64 mg/kg) was chosen based on our previous study showing the development of rapid-onset sensitization in mice (Wuo-Silva et al., 2016).

### Behavioral Test: Open-Field

As previously described, locomotor activity was measured in the open field arena (Chinen et al., 2006; Wuo-Silva et al., 2016). The open-field apparatus consisted of a circular wooden box (40 cm in diameter and 50 cm high) with an open top and a floor divided into 19 approximately similar regions delimited by three concentric circles of different radii (8, 14, and 20 cm) intersected by radial line segments. Using hand-operated counters and stopwatches, the locomotor activity (total number of entrances into any floor unit, i.e., number of crossings) was measured by an observer who was blind to the treatment allocation during a 10-min session. This interval has been proven effective in detecting behavioral sensitization induced by repeated treatment or a single injection of modafinil in mice (Wuo-Silva et al., 2011, 2016).

### Experimental Procedures

#### Experiment 1: Effects of the Dopamine D1 Receptor Antagonist SCH 23390 on the Acute Locomotor Stimulant Effect of Modafinil

Eighty mice were habituated to the open field (10-min sessions) and to the injection procedure for three consecutive days, and their locomotor activity was measured on day 3. After the habituation phase, animals were allocated into eight groups of comparable baseline locomotor activity (*n* = 10). On the 4th day, animals received an i.p. injection of either vehicle solution or 0.003, 0.006, or 0.01 mg/kg SCH 23390 followed by an i.p. injection of vehicle (Veh, SCH0.003, SCH0.006, and SCH0.01 groups) or 64 mg/kg modafinil (Mod, SCH0.003+Mod, SCH0.006+Mod, and SCH0.01+Mod groups) 30 min later. Thirty min after the 2nd injection, animals were placed individually in the open field and their locomotor activity was measured for 10 min.

#### Experiment 2: Effects of the Dopamine D2 Receptor Antagonist Sulpiride on the Acute Locomotor Stimulant Effect of Modafinil

Seventy-six mice were habituated to the open field (10-min sessions) and to the injection procedure for three consecutive days, and their locomotor activity was measured on day 3. After the habituation phase, animals were allocated into eight groups of comparable baseline locomotor activity (*n* = 9–10). On the 4th day, animals received an i.p. injection of either vehicle solution or 25, 50, or 100 mg/kg sulpiride followed by an i.p. injection of vehicle (Veh, Mod, Sulp25, Sulp50, and Sulp100 groups) or 64 mg/kg modafinil (Mod, Sulp25+Mod, Sulp50+Mod, and Sulp100+Mod groups) 30 min later. Thirty min after the 2nd injection, animals were placed individually in the open field and their locomotor activity was measured for 10 min.

#### Experiment 3: Effects of SCH 23390 or Sulpiride on the Development of Rapid-Onset Behavioral Sensitization Induced by Modafinil

The doses of SCH 23390 and sulpiride for Experiments 3 and 4 were chosen based on the results from Experiments 1 and 2. For SCH 23390, the dose of 0.003 mg/kg was chosen because it was the lowest dose to attenuate the stimulant locomotor effects of modafinil, yet did not modify spontaneous locomotion. With respect to the effects of SCH 23390 alone, there was only a non-significant trend toward a reduction in spontaneous locomotion at the highest dose (0.01 mg/kg). The dose of 50 mg/kg sulpiride was chosen because it produced no effects on modafinil-induced locomotor stimulation or on animals’ spontaneous locomotion.

The 4-h interval between the priming and the challenge injections was determined while characterizing the rapid-onset locomotor sensitization to modafinil (Wuo-Silva et al., 2016). With a 4-h interval, the priming injection of 64 mg/kg modafinil is no longer on board, thereby avoiding false positive results during the challenge session. The priming injection was administered in the morning, between 9:00 and 12:00, and the challenge injection was administered in the afternoon, between 13:00 and 15:00.

Fifty-nine mice were habituated to the open field (10-min sessions) and to the injection procedure for three consecutive days, and their locomotor activity was measured on day 3. After the habituation phase, animals were allocated into five groups of comparable baseline locomotor activity (*n* = 11–12). On the 4th day, animals received an i.p. pretreatment of vehicle solution (three control groups), 0.003 mg/kg SCH 23390 or 50 mg/kg sulpiride followed by an i.p. priming injection of vehicle (two control groups) or 64 mg/kg modafinil (all other groups) 30 min later. Immediately after the injections, animals were returned to their home cages. Four hours after the priming injections, animals received an i.p. challenge injection of vehicle (Veh+Veh-Veh group) or 64 mg/kg modafinil (Veh+Veh-Mod, Veh+Mod-Mod, SCH+Mod-Mod, and Sulp+Mod-Mod groups). Thirty min later, animals were placed individually in the open field and their locomotor activity was measured for 10 min.

#### Experiment 4: Effects of SCH 23390 or Sulpiride on the Expression of Rapid-Onset Behavioral Sensitization Induced by Modafinil

Sixty mice were habituated to the open field (10-min sessions) and to the injection procedure for three consecutive days, and their locomotor activity was measured on day 3. After the habituation phase, animals were allocated into five groups of comparable baseline locomotor activity (*n* = 12). On the 4th day, animals received an i.p. priming injection of vehicle solution (two control groups) or 64 mg/kg modafinil (three modafinil groups). Immediately after the injections, animals were returned to their home cages. Four hours after the priming injections, animals received an i.p. injection of either vehicle (two control groups and one modafinil group), 0.003 mg/kg SCH 23390 or 50 mg/kg sulpiride followed by an i.p. injection of vehicle (Veh-Veh+Veh group) or 64 mg/kg modafinil (Veh-Veh+Mod, Mod-Veh+Mod, Mod-SCH+Mod, and Mod-Sulp+Mod groups) 30 min later. Thirty min after the challenge injections, mice were placed individually in the open field and their locomotor activity was measured for 10 min.

### Statistical Analysis

The locomotor responses were checked for normality (Shapiro–Wilk test) and homogeneity of variances (Levene’s test), which validated the use of parametric tests. Multiple comparisons were performed using one-, two-, or three-way analysis of variance (ANOVA) using the General Linear Model test, with repeated measures (RM) or not, and Tukey’s *post hoc* test when necessary. A probability of *p* < 0.05 was considered a statistically significant difference.

## Results

### Experiment 1: Effects of the Dopamine D1 Receptor Antagonist SCH 23390 on Modafinil-Induced Locomotor Stimulation

**FIGURE 1:**
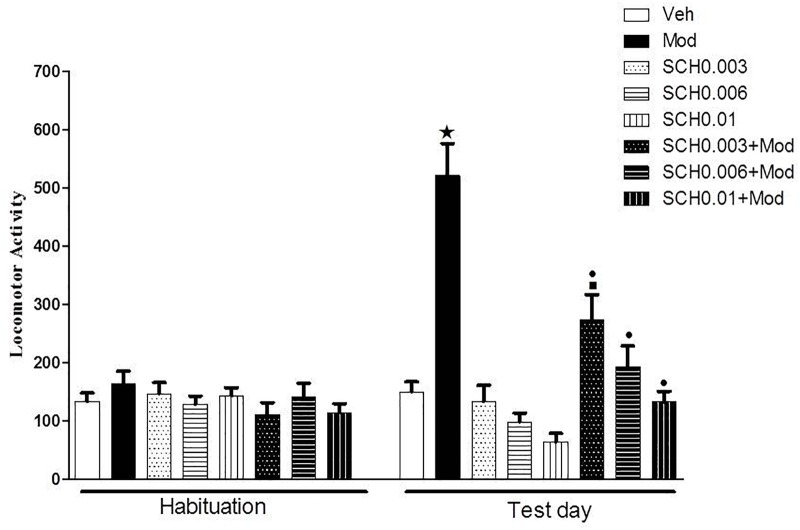
Locomotor activity (indicated as number of crossings) of mice on the 3rd day of habituation and on the test session. Animals received an i.p. injection of either vehicle solution or 0.003, 0.006, or 0.01 mg/kg SCH 23390 followed by an i.p. injection of vehicle (Veh, SCH0.003, SCH0.006 and SCH0.01 groups) or 64 mg/kg modafinil (Mod, SCH0.003+Mod, SCH0.006+Mod, and SCH0.01+Mod groups) 30 min later. Thirty min after the 2nd injection, the locomotor activity was measured for 10 min in the open field. SCH 23390 induced a dose-dependent attenuation of modafinil-induced locomotor stimulant effects, but did not alter spontaneous locomotion. Data are reported as means ± SEM (*n* = 10). ^
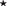
^*P* < 0.05 compared with the Veh group. ^
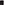
^*P* < 0.05 compared with the respective control group, which received SCH 23390 only. ^
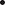
^*P* < 0.05 compared with the Mod group.

On the test day (day 4), two-way ANOVA revealed a significant SCH 23390 × modafinil interaction [*F*(3.76) = 9.6, *p* < 0.05] (Figure 1). Tukey’s *post hoc* test revealed that SCH 23390 alone did not modify spontaneous locomotion. Animals treated with 64 mg/kg modafinil alone (Mod) showed a significant increase in locomotor activity when compared to animals treated with vehicle (Veh), demonstrating the locomotor stimulant effect induced by this dose of modafinil. SCH 23390 induced a dose-dependent attenuation of modafinil-induced hyperlocomotion, with the groups treated with the middle and high doses of SCH 23390 not differing from their respective control groups.

### Experiment 2: Effects of the Dopamine D2 Receptor Antagonist Sulpiride on Modafinil-Induced Locomotor Stimulation

**FIGURE 2:**
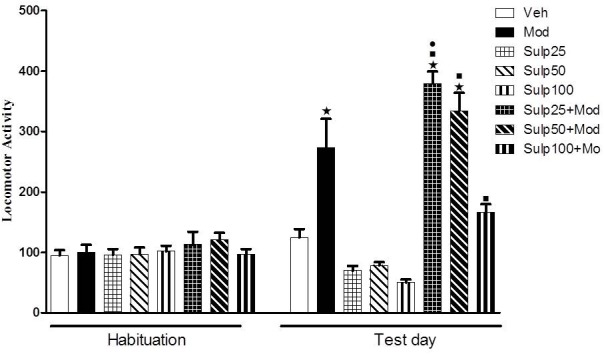
Locomotor activity (indicated as number of crossings) of mice on the 3rd day of habituation and on the test session. Animals received an i.p. injection of either vehicle solution or 25, 50, or 100 mg/kg sulpiride followed by an i.p. injection of vehicle (Veh, Mod, Sulp25, Sulp50, and Sulp100 groups) or 64 mg/kg modafinil (Mod, Sulp25+Mod, Sulp50+Mod, and Sulp100+Mod groups) 30 min later. Thirty min after the 2nd injection, the locomotor activity was measured for 10 min in the open field. The lowest dose of sulpiride increased, while the highest dose of sulpiride attenuated, modafinil-induced locomotor stimulant effects. None of the doses of sulpiride altered spontaneous locomotion. Data are reported as means ± SEM (*n* = 9–10). ^
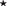
^*P* < 0.05 compared with the Veh group. ^
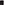
^*P* < 0.05 compared with the respective control group, which received sulpiride only. ^
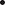
^*P* < 0.05 compared with the Mod group.

On the test day (day 4), two-way ANOVA revealed a significant sulpiride × modafinil interaction [*F*(3,72) = 8.3, *p* < 0.05] (Figure 2). Tukey’s *post hoc* test revealed that none of the doses of sulpiride modified spontaneous locomotion. Animals treated with 64 mg/kg modafinil alone (Mod) showed a significant increase in locomotor activity when compared to animals treated with vehicle (Veh), demonstrating again the locomotor stimulant effect induced by this dose of modafinil. Interestingly, the Sulp25+Mod group showed a significant increase in locomotor activity compared to the Mod group, indicating that pre-treatment with 25 mg/kg sulpiride potentiated modafinil-induced hyperlocomotion. Sulpiride at 50 mg/kg did not modify the stimulant effects of modafinil on locomotor activity. Finally, the highest dose of sulpiride (100 mg/kg) attenuated the locomotor stimulant effect of modafinil, as locomotion of the Sulp100+Mod group did not differ statistically from that of their respective control group.

### Experiment 3: Effects of SCH 23390 or Sulpiride on the Development of Rapid-Onset Behavioral Sensitization Induced by Modafinil

**FIGURE 3:**
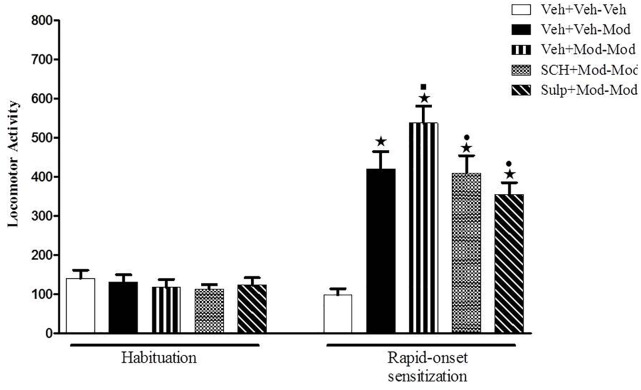
Locomotor activity (indicated as number of crossings) of mice on the 3rd day of habituation and on the challenge session of the rapid-onset behavioral sensitization test. Animals received an i.p. pretreatment injection of vehicle solution (three control groups), 0.003 mg/kg SCH 23390 or 50 mg/kg sulpiride followed by an i.p. priming injection of vehicle (two control groups) or 64 mg/kg modafinil (all other groups) 30 min later. Immediately after the injections, animals were returned to their home cages. Four hours after the priming injections, animals received an i.p. challenge injection of vehicle (Veh+Veh–Veh group) or 64 mg/kg modafinil (Veh+Veh–Mod, Veh+Mod–Mod, SCH+Mod–Mod, and Sulp+Mod–Mod groups). Thirty min later, the locomotor activity was measured for 10 min in the open field. Both dopaminergic antagonists prevented the development of rapid-onset behavioral sensitization to modafinil. Data are reported as means ± SEM (*n* = 11–12). ^
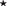
^*P* < 0.05 compared with the Veh+Veh–Veh group. ^
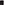
^*P* < 0.05 compared with the Veh+Veh–Mod group. ^
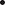
^*P* < 0.05 compared with the Veh+Mod–Mod group.

On the sensitization test (day 4), significant differences between groups were detected by one-way ANOVA [*F*(4,54) = 54.3, *p* < 0.05] (Figure 3). Tukey’s *post hoc* test revealed that the animals that were treated acutely with modafinil (priming injection of vehicle and challenge injection of modafinil – Veh-Mod) presented a significant increase in locomotion when compared with the Veh-Veh control group, demonstrating the locomotor stimulant effects of modafinil. The animals that received a priming and a challenge injection of 64 mg/kg modafinil (Mod-Mod group) presented significantly greater locomotor activity when compared to their respective control group that was treated initially with vehicle and challenged with 64 mg/kg modafinil (Veh-Mod), characterizing the development of rapid-onset behavioral sensitization to modafinil. Notably, the animals that were treated with the D1 antagonist SCH 23390 or the D2 antagonist sulpiride combined with the priming injection of modafinil (SCH+Mod-Mod and Sulp+Mod-Mod) showed a significant decrease in the locomotor activity during the challenge session when compared to the sensitized group (Mod-Mod). Levels of locomotor activity presented by the SCH+Mod-Mod and Sulp+Mod-Mod groups were comparable to those presented by the group that was receiving modafinil for the first time (Veh-Mod). Taken together, these data demonstrate that both dopaminergic antagonists were able to prevent the development of rapid-onset behavioral sensitization to modafinil.

### Experiment 4: Effects of SCH 23390 or Sulpiride on the Expression of Rapid-Onset Behavioral Sensitization Induced by Modafinil

**FIGURE 4:**
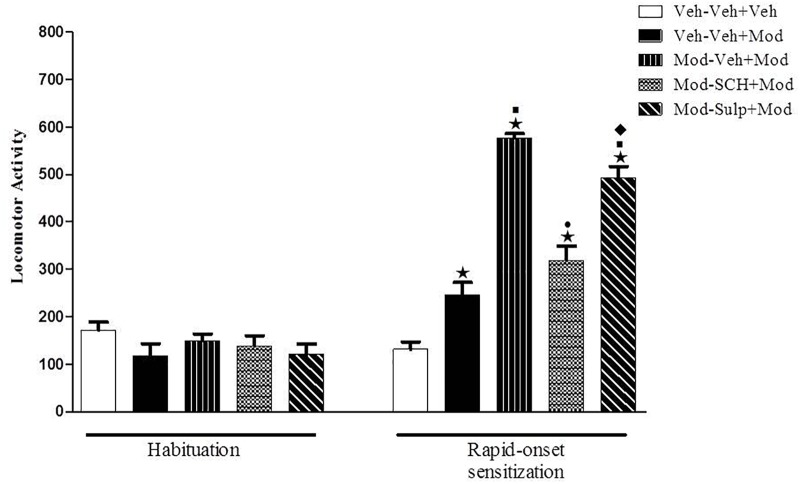
Locomotor activity (indicated as number of crossings) of mice on the 3rd day of habituation and on the challenge session of the rapid-onset behavioral sensitization test. Animals received an i.p. priming injection of vehicle solution (two control groups) or 64 mg/kg modafinil (three modafinil groups). Immediately after the injections, animals were returned to their home cages. Four hours after the priming injections, animals received an i.p. injection of either vehicle (two control groups and one modafinil group), 0.003 mg/kg SCH 23390 or 50 mg/kg sulpiride followed by an i.p. injection of vehicle (Veh–Veh+Veh group) or 64 mg/kg modafinil (Veh–Veh+Mod, Mod–Veh+Mod, Mod–SCH+Mod, and Mod–Sulp+Mod groups) 30 min later. Thirty min after the challenge injection, the locomotor activity was measured for 10 min in the open field. Only SCH 23390 prevented the expression of modafinil-induced rapid-onset locomotor sensitization. Data are reported as means ± SEM (*n* = 12). ^
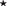
^*P* < 0.05 compared with the Veh–Veh+Veh group. ^
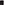
^*P* < 0.05 compared with the Veh–Veh+Mod group. ^
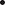
^*P* < 0.05 compared with the Mod–Veh+Mod group. ^
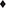
^*P* < 0.05 compared with the Mod–SCH+Mod group.

One-way ANOVA revealed significant differences between groups on the sensitization test day (day 4) [*F*(4,55) = 65.1, *p* < 0.05] (Figure 4). Tukey’s *post hoc* test revealed that a single acute injection of 64 mg/kg modafinil induced hyperlocomotion in mice, as seen by an increased locomotor activity in the Veh-Mod group compared with the Veh-Veh control group. The animals that were primed and challenged with 64 mg/kg modafinil (Mod-Mod group) presented significantly greater locomotor activity when compared to the Veh-Mod group, characterizing the development of rapid-onset behavioral sensitization to modafinil. Interestingly, only SCH 23390 was able to prevent the expression of modafinil-induced rapid-onset locomotor sensitization, as locomotor activity of the Mod-SCH+Mod, but not of the Mod-Sulp+Mod group, was reduced compared to the locomotor activity of the Mod-Mod group.

In both Experiments 3 and 4, we did not observe stereotypical behavior in mice 4 h after the priming-injection of modafinil. This suggests that, although a 4-h interval was sufficient to induce locomotor sensitization to modafinil, this interval did not lead to the development of stereotypical behavior.

## Discussion

The present study shows the effects of administration of dopamine D1 and D2 receptor antagonists (SCH 23390 and sulpiride, respectively) on acute modafinil-induced hyperlocomotion and on the development and expression of rapid-onset locomotor sensitization to modafinil in mice. Our findings show that both dopamine antagonists blocked the acute locomotor effects of modafinil in a dose-dependent manner and prevented the development of modafinil-induced rapid-onset sensitization. On the other hand, only SCH 23390 was effective at inhibiting the expression of locomotor sensitization to modafinil. These findings suggest that both dopamine D1 and D2 receptors are involved in the acute hyperlocomotor effects of modafinil and in the development of modafinil-induced rapid-onset sensitization, while D1, but not D2, dopamine receptors seem to be associated with the expression of this type of sensitization.

Regarding the effects of the dopaminergic antagonists on acute locomotor stimulation induced by modafinil, all doses of SCH 23390 attenuated modafinil-induced hyperlocomotion. On the other hand, the lowest dose of sulpiride significantly potentiated, whereas the highest dose significantly attenuated, modafinil-induced locomotor stimulant effects. Our data related to the effects of sulpiride are in agreement with the study from Dias et al. (2010), which showed that 10 mg/kg and 30 mg/kg sulpiride potentiated the locomotor stimulant effects of the D1/D2 agonist apomorphine in rats, while 100 mg/kg sulpiride attenuated apomorphine-induced hyperlocomotion. Importantly, sulpiride has a preferential affinity for D2 autoreceptors (Carey et al., 2008). Low doses of sulpiride preferentially block D2 autoreceptors, leading to an increase in dopamine release, which can potentiate the locomotor stimulant effects induced by dopaminergic agonists (Carey et al., 2008). In contrast, at higher doses, sulpiride binds not only to D2 autoreceptors, but also to post-synaptic D2 receptors, which could attenuate the hyperlocomotor effects of dopaminergic agonists (Carey et al., 2004). Because modafinil promotes the increase of extracellular dopamine levels via an indirect pathway and has been proposed to be a D2 receptor agonist (Korotkova et al., 2007), the varied effects of sulpiride on modafinil-induced locomotor stimulation may be due to the dose-dependent effects of sulpiride on either type of dopamine D2 receptors.

To the best of our knowledge, this is the first study describing a role for dopamine receptors in the induction and expression of rapid-onset behavioral sensitization to modafinil. Previous studies have demonstrated the participation of dopamine receptors in both the rewarding effects and behavioral sensitization induced by repeated administration of modafinil (Chang et al., 2010; Nguyen et al., 2011). Rats treated for 10 days with apomorphine, a D1/D2 receptor agonist, expressed behavioral sensitization to a challenge injection of 64 mg/kg modafinil (Chang et al., 2010). Autoradiography assays also indicated that conditioned place preference induced by 125 mg/kg modafinil in mice produced an increase in D1 receptor binding in the caudate putamen, the nucleus accumbens and the substantia nigra, and a decrease in D2 receptor binding in the caudate putamen and the nucleus accumbens (Nguyen et al., 2011). Administration of D1 and D2 receptor antagonists (SCH 23390 and raclopride) prior to the administration of low doses (22.5 and 45 mg/kg) of modafinil has also been shown to abolish the wake-promoting effects of modafinil (Qu et al., 2008). Modafinil also had no alertness-inducing effects in D2 knockout mice pretreated with the D1 antagonist SCH 23390 (Qu et al., 2008; Young, 2009). Recently, Alam and Choudhary (2018) have shown that behavioral sensitization induced by repeated treatment with 64 mg/kg modafinil in rats could be reduced by a challenge injection of the D1/D2 receptor antagonist haloperidol. These findings suggest that the mechanisms involved in the behavioral sensitization induced by repeated administration of modafinil are similar to those previously shown for other psychostimulant drugs.

Importantly, our results corroborate a study by Kuczenski and Segal (1999a), which evaluated the effects of dopaminergic antagonists on rapid-onset behavioral sensitization to amphetamine in rats. In this study, the authors demonstrated that administration of the D1 receptor antagonist SCH 23390 or the D1/D2 receptor antagonist haloperidol prior to a priming injection of 4 mg/kg amphetamine inhibited the development of amphetamine-induced sensitization to stereotyped behavior when a challenge injection of amphetamine was administered 5 h later. Together with our findings, such results indicate that rapid changes in the sensitivity of dopamine receptors occur following a single administration of a high dose of psychostimulants. These changes may explain the mechanisms involved in binge patterns of drug abuse, i.e., when high doses of a drug are used in a short period of time. In a later study, the same authors (Kuczenski and Segal, 1999b) demonstrated that priming injections of D1 or D2 receptor agonists (SKF 82958 and quinpirole, respectively) resulted in enhanced stereotypical behaviors in rats when a subthreshold dose of amphetamine was administered a few hours later. Those findings further confirm the importance of both dopamine D1 and D2 receptors for the development of rapid-onset sensitization to amphetamine-induced stereotyped behavior.

A limitation of preclinical studies with modafinil, particularly rodent studies, is that the doses of modafinil used in the clinics are much lower (100–200 mg or 1–3 mg/kg) than those commonly used in laboratory animals (30–300 mg/kg), contributing to the difficulty in translating preclinical findings to humans. The use of lower doses in the clinics might also contribute to the lack of modafinil abuse described in clinical trials. However, our and other findings emphasize that when taken at higher doses, such as in recreational use, modafinil could have abuse potential in humans. In fact, two recent case report studies have described that patients who started treatment with modafinil with increasing doses reported withdrawal symptoms similar to those experienced during abstinence of psychostimulant drugs upon discontinuation of modafinil (Swapnajeet et al., 2016; Alacam et al., 2018).

Importantly, the dopaminergic system also seems to be involved in the wake-promoting effects of modafinil. Wisor et al. (2001) have demonstrated that modafinil does not induce wakefulness in DAT knockout mice. As previously mentioned, D1 and D2 receptors have also been shown to play an important role in the wake-promoting effects of modafinil (Qu et al., 2008). Therefore, one could argue that the changes in locomotor activity observed in the present study in mice treated with modafinil could be related to changes in modafinil-induced alertness. However, studies have shown that the locomotor stimulant effects of psychostimulant drugs do not seem to be related to their wake-promoting effects. Shelton et al. (1995) demonstrated that i.v. administration of 10 mg/kg modafinil or 200 μg/kg amphetamine in narcoleptic dogs increased wakefulness, but did not alter locomotor activity. Likewise, Edgar and Seidel (1997) have shown that different doses of modafinil increased alertness but did not alter the locomotor activity of rats. Such findings suggest that the wake-promoting effects of modafinil may be dissociated from its effect on locomotor activity. Future studies are needed to clarify this idea.

Although the behavioral effects of modafinil seem to resemble those of other psychostimulants, distinct mechanisms of action have been proposed between modafinil and psychostimulant drugs. For this reason, modafinil has been considered a drug with low abuse potential that could be used as a pharmacotherapy capable of reversing the neuroadaptations caused by the chronic use of cocaine and other drugs of abuse (Minzenberg and Carter, 2008; Mereu et al., 2013). Nonetheless, our studies have demonstrated that modafinil induces behavioral sensitization under the repeated treatment protocol, under the two-injection protocol (Wuo-Silva et al., 2011) and under the rapid-onset paradigm (Wuo-Silva et al., 2016). We have also demonstrated that modafinil induces cross-sensitization with the behavioral effects of cocaine in all those paradigms (Wuo-Silva et al., 2011, 2016). Further studies have also shown that modafinil has potential for abuse in both rodents and humans, as it induces conditioned place preference in rats (Shuman et al., 2012) and exerts reinforcing and rewarding effects and increases incentive salience in humans (Stoops et al., 2005; Smart et al., 2013; Funayama et al., 2014). These results demonstrate the importance of understanding the mechanisms involved in animal models of modafinil abuse. The results of the present study strengthen and complement those findings by showing the participation of dopamine D1 and D2 receptors in the induction and/or expression of rapid-onset behavioral sensitization to modafinil. The present findings suggest that there may be rapid changes in the sensitivity of these receptors shortly after a single modafinil injection.

## Author Contributions

RW-S, DF-L, RF-F, and BL were responsible for the study concept and design. RW-S, DF-L, BF, AH, RS-B, EM-K, TY, and LL-S contributed to the acquisition of animal data. RW-S, DF-L, EM, LB, RF-F, and BL assisted with data analysis and interpretation of findings. RW-S, DF-L, LB, and BL drafted the manuscript. All authors critically reviewed the content and approved the final version of the manuscript for publication.

## Conflict of Interest Statement

The authors declare that the research was conducted in the absence of any commercial or financial relationships that could be construed as a potential conflict of interest.
